# Patient satisfaction with nurse-delivery primary health care services in Free State and Gauteng provinces, South Africa: A comparative study

**DOI:** 10.4102/phcfm.v9i1.1262

**Published:** 2017-04-28

**Authors:** Wilfred N. Nunu, Pascalia O. Munyewende

**Affiliations:** 1 School of Public Health, Faculty of Health Sciences, University of the Witwatersrand, South Africa; 2 Department of Environmental Science and Health, Faculty of Applied Sciences, National University of Science and Technology, Zimbabwe; 3 Centre for Health Policy and Medical Research Council Health Policy Research Group, School of Public Health, Faculty of Health Sciences, University of Witwatersrand, South Africa

## Abstract

**Background:**

The majority of health care users in South Africa utilise primary health care (PHC) services where these services are free at the point of entry. There is a dearth of knowledge on the factors influencing patient satisfaction with PHC clinic services.

**Aim:**

This study compared patient satisfaction with PHC services in the Free State (FS) and Gauteng (GP) provinces

**Setting:**

Secondary data analysis was conducted on a cross-sectional survey obtained from the Research on the State of Nursing Project run by the Centre for Health Policy in 2012.

**Methods:**

A pre-tested satisfaction survey questionnaire with questions on facility evaluation, experience with providers and receipt of medication was administered to 1110 systematically randomly sampled adult patients attending antiretroviral, hypertension, diabetes and tuberculosis services.

**Results:**

Of 1110 respondents, 1096 responded to the patient satisfaction survey signifying a 98.8% response rate. Over 60% of respondents were women in both provinces. Over 90% of patients were satisfied with PHC services in both provinces. Factors associated with satisfaction in GP and FS were time spent waiting for consultation, nurses listened, being given information on condition and being treated politely. Having privacy respected came out as a significant factor in FS.

**Conclusions:**

High levels of satisfaction with PHC services were experienced by study participants in both provinces. Satisfied patients adhere to treatment plans and have better health-seeking behaviour, which translates to improved clinical outcomes. Therefore, nurses should continue listening, respecting and treating their patients with politeness, and also implement efficient work schedules to reduce patient waiting times.

## Introduction

Globally, patient satisfaction is considered an important aspect in shaping the quality of health system reforms and health care service delivery.^1,2,3^ In Germany and France, it is compulsory for facilities to conduct patient satisfaction surveys in line with their policies to assess the performance of their respective health systems.^2,4^ In Europe and the United States, patient satisfaction research has been influential in providing evidence for policymakers to improve health system performance in hospitals.^4,5,6,7,8^ In South Africa, limited research studies have focused on patient satisfaction particularly at the primary health care (PHC) level.^9^ PHC level refers to the first point of call in the South African health system offering basic, curative and preventative care services.^9,10^ It excludes services that are complicated and require specialised care, at the hospital level for example.^7^ PHC refers to the essential services that are offered by health facilities in the community. They mostly focus on integrated management of childhood illnesses (IMCI), sexually transmitted infections (STIs) or HIV or AIDS, tuberculosis (TB), reproductive health (ante-natal care, family planning and maternity), mental health, chronic diseases (hypertension, diabetes and asthma), trauma and injuries and disabilities.^9,11,12,13^ Patient surveys assist in understanding patient behaviour, mostly that patients with high levels of satisfaction are more likely to adhere to their treatment regimens as well as to follow health worker advice on improving their conditions.^1,3^

However, where patients’ satisfaction research has been conducted, the literature highlights that most of these studies show a lack of agreement on the standard methodologies that can be used to measure it.^4,7,14^ Researchers also lack agreement on the type of satisfaction that is most important for patients, whether it is their satisfaction with the health care system in general or with a particular facility.^8^ Different countries have different contextual settings that could influence failure or success of a methodology to be used to assess patient satisfaction.^15^ It should be understood that patient satisfaction surveys have to take into consideration contextual factors that could be privy to that area of interest to ensure clear understanding of matters.^15^ Ultimately, the literature highlights the importance of the different kinds of patient satisfaction where results show that the quality of care and having happier patients who adhere to treatment is often the result of being satisfied with health care services.^7,8^ Using comparative research methods on patient satisfaction is important in improving the performance of the health care system, reducing costs and for the successful implementation of health system reforms such as PHC re-engineering and the National Health Insurance (NHI). Policymakers are given the opportunity to make informed decisions that could be unveiled through conducting these kinds of patient satisfaction surveys that highlight differences in different provincial settings.^15^

There is a dearth of patient satisfaction surveys that compare patient satisfaction with PHC services utilisation in Gauteng (GP) and Free State (FS) provinces.^16^ Given renewed government commitment on health systems reform through PHC re-engineering and the impending NHI, it becomes critical to have patient satisfaction surveys giving a glimpse of the direction health policy should take.^6^

The South African government has reiterated the importance of PHC making services free at the point of entry and revitalising services at that level.^17^ Conducting patient satisfaction surveys will provide a window of opportunity through which the quality of programmes can be evaluated and measured. Analysing the differences and similarities in patient satisfaction in GP and FS will provide an insight into factors that have to be taken into account for both PHC re-engineering and NHI reforms. Over 60% of health facilities in GP are owned by the local authority, whereas all the facilities in FS are owned by the provincial government. This study sought to compare and contrast patient satisfaction with PHC services in the FS and GP provinces. The study also identified and analysed critical predictors of patient satisfaction with PHC services in both these provinces.

## Materials and methods

### Study design and setting

Secondary data analysis was conducted on data that were obtained from a cross-sectional survey on the Research on the State of Nursing (RESON) that was conducted by the Centre for Health Policy (CHP) during July to September 2012.^18^ The survey (RESON) was conducted on day patients attending sampled PHC clinics representing the municipal districts in GP and FS provinces.^18^ GP is the most populated province in South Africa with a population estimate of 11.1 million representing 22% of the South African population.^18^ GP is highly urbanised with 97% of its population living in urban areas and only 3% living in rural areas.^17,18^ In a large proportion of people in GP (as compared to other provinces), 25% have private health insurance leaving 75% of the population relying on government-operated facilities. FS is largely a rural and agricultural province with an estimated population of about 2.8 million people.^19^ Of this population, only 13% have private health insurance meaning the remaining 87% rely on government-operated facilities for their health care needs.^19^ The province is underdeveloped when compared to the GP.^19^

### Study population and sampling

The primary study population consisted of patients visiting eight-hour day clinics in each province.^18^ Eight-hour clinics are defined as those clinics that offer services during the day for five days a week.^6,18^ The primary study focused on adult chronic patients receiving antiretroviral (ART), hypertension, diabetes and tuberculosis services in these PHC clinics.^18^ The inclusion criteria were patients who had a history with the clinic and were in possession of a patient retained record or caregivers of children attending child health and well-baby clinics.^18^ This was done so as to get individuals who would have used the services more than once so as to avoid subjectivity in their responses. Considering that this study was based on secondary data analysis, we found that the methods followed by the RESON researchers in the sample size calculation were appropriate. A two-stage sampling technique was followed:

The first step involved generating a list of the eight-hour clinics in each province. Proportional random sampling according to the number of districts in each municipality was done.^18^ The sample was calculated by assuming the number of satisfied PHC nursing managers managing individual clinics. This was pegged at 50% with an assumed 10% precision. The significance level was set at 5% with a confidence interval of 95% (calculated using Epi Info, Version 6, Centre for Disease Control and Prevention, Atlanta, GA, USA). The desired sample size was 96 clinics. Sixteen per cent^15^ of the PHC clinics were added to the total sample in case of refusals or other reasons. Ultimately, 111 clinics (GP 60%, *n* = 67 and FS 40%, *n* = 44) constituted the total study sample.^20^

The second step of the sampling involved patients. Patients were selected using systematic random sampling to obtain five adults and five children from each clinic.^18^ Primary researchers would find out the number of patients seen per day (adults and children). That number would then be divided by five to determine the interval of systematic random sampling. A total of 1096 respondents were obtained against the 1110 targeted resulting in a response rate of 98.8%. There were 627 from GP and 469 from FS.^18^ Fourteen respondents were excluded because they did not meet the study selection recruitment criteria.

A structured questionnaire was used in the primary study.^18^ Questions were asked in stages. Firstly, whilst the patient was waiting for their medical consultation with the clinic staff; secondly, after the consultations; and thirdly, before exiting the clinic with additional questions asking patients to comment on the PHC services given generally.^18^ These were face-to-face interviews conducted on patients mainly in English.^18^ In cases where respondents had challenges in understanding English, trained data collectors would interview the patient in his or her language of choice.^18^ The patient satisfaction survey was piloted in three clinics similar to the study sample, and no adjustments were made.^18^

### Data analysis

#### Data management

Data were available in an Excel spreadsheet and were then imported into STATA Version 13 for analysis. From the secondary data set, demographic and other variables relating to patients’ satisfaction with PHC services were selected, and these were province, sex, length of clinic usage, cleanliness of facility, time spent, privacy, availability of prescribed medicines, treatment (respect) and encouraging others to visit clinic. The data were checked for internal consistency using the Cronbach’s scale, and a value of 0.938 was obtained symbolising 93.8% reliability.

#### Analysis

A scoring scale was developed to measure levels of patient satisfaction using data obtained from 17 binary questions that were used in the primary survey responses, which were aggregated to generate the scores Yes = 1 and No = 0. Three categories were developed (dissatisfied – between 0 and 6 yeses, indifferent – between 7 and 11 yeses, and satisfied – between 12 and 17 yeses) as adapted from Spooren and Smith.^21,22^ Chi-squared (*χ*^2^) tests were conducted to determine whether there was significant difference on the distribution of respondents under these three categories and comparison made between the two provinces. Weighting of the data was done to accommodate provincial differentiation using the Kruskal–Wallis commands of weighting on STATA.

Cross tabulations were done to ascertain how different factors influenced satisfaction using ‘willingness to encourage friends and family to use the clinics’ as a proxy of being satisfied with the services offered. Multiple regressions were performed to examine relationships between multiple factors and their effect on patient satisfaction using ‘willingness to refer their friends and family to the same clinic they were attended to’ as a proxy for satisfaction (see Appendix 1). This proxy enables patients to give a holistic overview of the whole treatment process thereby eliminating the element of subjectivity with different stages.^16,23^

## Results

There were more women than men in this sample for both provinces (GP 62% (393) *n* = 627; FS 66% (311) *n* = 469). During the period under review, that is, July to September 2012, a large percentage in FS on average (87%) walked to their facilities as compared with GP (65%). Patients in FS utilised about 1 rand on average on transport, whereas in GP the average amount paid for transport was 3.6 rand. During this period, the rand was trading at 8.16 to 8.31 to the US dollar. These findings are presented in Table 1 and Table 2.

**TABLE 1:** Respondents, demographics and clinic accessibility and utilisation.

Variables	Gauteng		Free State	
	
	Number	Percentage	Number	Percentage
Sex				
Male	231	36.80	158	33.70
Female	393	62.70	311	66.30
Missing	3	0.50	0	0.00
Total	627	100.00	469	100.00
First time to use the clinic				
Yes	51	8.10	16	3.40
No	570	90.90	448	95.50
Missing	6	1.00	5	1.10
Total	627	100.00	469	100.00
Get to the clinic				
Walk	398	63.48	392	83.60
Taxi	138	22.00	44	9.40
Lift with family members	19	3.00	11	2.30
Own car	49	7.80	16	3.40
Others	22	3.50	6	1.30
Missing	1	0.20	0	0.00
Total	627	100.00	469	100.00
Pay for transport				
Yes	155	24.70	53	11.30
No	418	66.70	341	72.70
Missing	54	8.60	75	16.00
Total	627	100.00	469	100.00
Stay in this catchment area				
Yes	548	87.40	439	93.60
No	72	11.50	27	5.80
Missing	7	1.10	3	0.60
Total	627	100.00	469	1000
Work in this area				
Yes	138	22.00	102	21.70
No	475	75.80	365	77.80
Missing	14	2.20	2	0.40
Total	627	100.00	469	100.00
Clinic clean				
Yes	556	88.70	426	90.80
No	65	10.40	38	8.10
Missing	6	1.00	5	1.10
Total	627	100.00	469	100.00
Toilets clean				
Yes	434	69.20	370	78.90
No	129	20.60	79	16.80
Missing	64	10.20	20	4.30
Total	627	100.00	469	100.00
Easy to find way in clinic				
Yes	578	92.20	445	94.90
No	40	6.40	22	4.70
Missing	9	1.40	2	0.40
Total	627	100.00	469	100.00
Opening hours convenient				
Yes	526	83.90	415	88.50
No	92	14.70	51	10.90
Missing	9	1.40	3	0.60
Total	627	100.00	469	100.00
Water available to drink				
Yes	563	89.80	432	92.10
No	54	8.60	37	7.90
Missing	10	1.60	0	0.00
Total	627	100.00	469	100.00
Bench to sit on				
Yes	608	97.00	454	96.80
No	15	2.40	12	2.60
Missing	4	0.60	3	0.60
Total	627	100.00	469	100.00
Noise acceptable				
Yes	522	83.30	353	75.30
No	102	16.30	115	24.50
Missing	3	0.50	1	0.20
Total	627	100.00	469	100.00

*Source:* Rispel 2008.^17^

**TABLE 2:** Amount paid for transport and time taken to get to clinics.

Variables	Gauteng	Free State
Mean	3.59	1.06
Standard deviation	7.65	3.61
Minutes to get to clinic		
Median	15	25
IQR	10–30	10–30
Hours to get to clinic		
Median	1	1
IQR	1–1	1–2
Months using this clinic		
Median	51	119.5
IQR	27.5–78	27–247

*Source:* Rispel 2008^17^

IQR, interquartile range.

**FIGURE 1:**
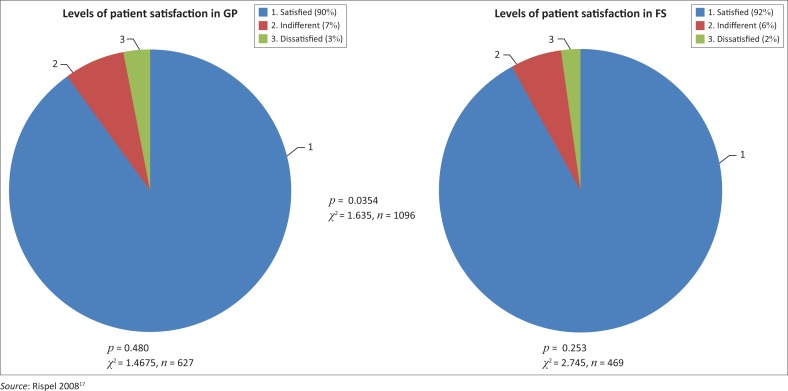
Levels of patient satisfaction with PHC in GP and FS.

Ninety per cent of respondents in clinics in GP were satisfied with the clinic they attended. In FS, 92% were satisfied with the clinic they attended. Chi-squared tests (*χ*^2^) were performed to ascertain whether or not there was difference between the provinces. As our *p*-value was less than 0.05, any difference in levels of patient satisfaction in the province was not statistically significant. The findings are summarised in Figure 1.

### Cross tabulations per province

Of the nine variables cross tabulated with this outcome (sex, how they got to clinic, time spent at the facilities, being listened to by nurses, knowing the name of nurse, having their privacy respected, being given information on condition, having medicines prescribed and being treated politely), in GP seven were significantly associated with the outcome at a *p*-value less than 0.05 (how they got to clinic, time spent at the facilities, being listened to by nurses, knowing the name of nurse, having their privacy respected, being given information on condition and being treated politely), and in FS five were significantly associated with the outcome at a *p*-value less than 0.05 (time spent at the facilities, having their privacy respected, being given information on condition, having medicines prescribed and being treated politely). Four variables showed significant provincial difference (get to clinic, knowing name of nurse, having privacy respected and having their medicines prescribed). These findings are presented in Table 3.

**TABLE 3:** Cross tabulations by province of factors that influenced satisfaction using ‘willingness to encourage friends and family to use the clinics’.

Factors	Gauteng		Free State		Comparison between the provinces
	
	No *n* (%)	Yes *n* (%)	No *n* (%)	Yes *n* (%)	
Sex					
Male	14 (6.3)	208 (93.7)	10 (6.5)	144 (93.5)	
Female	30 (7.8)	357 (92.3)	16 (5.3)	289 (94.8)	
Total	44	565	26	433	
*p*	0.5070		0.5850		0.672
Get to clinic					
Walk	32 (8.3)	356 (91.7)	23 (5.9)	364 (94.1)	
Taxi	7 (5.2)	128 (94.8)	3 (7.1)	39 (92.9)	
Lift with family members	4 (21.1)	15 (78.9)	0	11 (100.0)	0.003
Own car	0	48 (100)	0	14 (100.0)	
Other	2 (9.5)	19 (90.5)	0	5 (100.0)	
Total	45	566	26	433	
*p*	0.0320		0.7300		
Time spent					
Too much	36 (13.6)	228 (86.4)	23 (12.7)	158 (87.3)	
Just right	9 (3)	287 (97)	3 (1.3)	234 (98.7)	
Too short	0	49 (100.0)	0	36 (100.0)	
Other	0	2 (100.0)	0	5 (100.0)	0.765
Total	45	566	26	433	
*p*	< 0.0001		< 0.0001		
Nurses listened					
No	19 (63.3)	11 (36.7)	15 (62.5)	9 (37.5)	
Yes	26 (4.5)	554 (95.5)	11 (2.5)	423 (97.5)	0.936
Total	45	565	26	432	
*p*-value	< 0.0001		< 0.0001		
Name of nurse					
No	33 (10.2)	292 (89.8)	9 (8.0)	103 (92.0)	
Yes	12 (4.2)	273 (95.8)	17 (4.9)	330 (95.1)	
Total	45	565	26	433	0.012
*p*	0.0050		0.2120		
Privacy respected					
No	9 (33.3)	18 (66.7)	7 (50.0)	7 (50.0)	
Yes	35 (6.1)	543 (93.9)	19 (4.3)	425 (95.7)	0.0534
Total	44	561	26	432	
*p*	< 0.0001		< 0.0001		
Information on condition					
No	21 (52.5)	19 (47.5)	16 (50.0)	16 (50.0)	
Yes	23 (4.0)	546 (96.0)	10 (2.4)	415 (97.6)	0.876
Total	44	565	26	431	
*p*	< 0.0001		< 0.0001		
Medicines prescribed					
No	7 (9.3)	68 (90.7)	5 (12.2)	36 (87.8)	
Yes	36 (6.8)	493 (93.2)	20 (4.8)	395 (95.2)	0.003
Total	43	561	25	431	
*p*	0.4260		0.0480		
Treated politely					
No	17 (85)	3 (15.0)	14 (73.7)	5 (26.3)	
Yes	28 (4.7)	563(95.3)	12 (2.7)	428 (97.3)	0.867
Total	45	566	26	433	
*p*	< 0.0001		< 0.0001		

*Source:* Rispel 2008^17^

*n*, number; *p, p*-value.

### Critical predictors of patient satisfaction

In GP, respondents who indicated that the amount of time spent at the clinic today was just right were about five times more likely to encourage a friend or relative that was sick to come to this clinic compared to those that replied that the time spent was too much. In FS, respondents who indicated that the amount of time spent at the clinic today was just right were about five times more likely to encourage a friend or relative that was sick to come to this clinic compared to those that replied that the time spent was too much. The findings are presented in Table 4.

**TABLE 4:** Provincial multiple logistic regressions.

Variable	Response	Gauteng			Free State			Comparison of the two provinces
	
		OR	95% CI	*p*	OR	95% CI	*p*	
Time spent	Too much	1	†	†	1	†	†	-
	Just right	4.5	1.68–12.12	0.0030	4.84	1.01–23.10	0.0480	0.0040
	Too short	†	†	†	†	†	†	-
	Other	†	†	†	†	†	†	-
Nurses listened	No	1	†	†	1	†	†	-
	Yes	6.18	2.02–18.89	0.0010	5.2	1.15–23.64	0.0330	0.0567
Information on condition	No	1	†	†	1	†	†	-
	Yes	8.14	3–22.08	< 0.0001	10.17	2.52–41.14	0.0010	0.0673
Treated politely	No	1	†	†	1	†	†	-
	Yes	22.95	5.3–99.36	< 0.0001	22.03	4.6–105.31	< 0.0001	0.0030
Privacy respected	No	†	†	†	1	†	†	-
	Yes	†	†	†	5.5	1.05–28.8	0.0440	0.9760

*Source:* Rispel 2008^17^

† , No estimates (sparse data).

*p, p*-value.; OR, odds ratio; CI, confidence interval.

## Ethical consideration

Ethics clearance for the secondary data analysis was sought from the University of the Witwatersrand Human Research Ethics Committee (Ethics Clearance Certificate Number: M141045).

## Discussion

Findings of the study pointed out that there is no significant difference in levels of patient satisfaction between the two provinces (GP and FS). Significant factors that influenced patient satisfaction in the two provinces were time spent at the clinic, being listened to by nurses, being treated politely. In FS, having privacy respected also came out as a significant factor. However, there were notable differences in the extent that these factors affected patient satisfaction in the two provinces, that is, being listened to by nurses, being given information on condition and having privacy respected had significantly higher impacts on patient satisfaction in FS as compared with GP. The remaining factors (time spent in clinics and being treated politely) showed similarities in the extent of influence on patient satisfaction. These factors could be improved and leveraged on to improve patient satisfaction, which in turn translates to better health outcomes for patients. Satisfied patients adhere to treatment regimens and this coupled with a good attitude may foster good health-seeking behaviour.^1,2^

The results highlighted that women were the majority of respondents in both provinces accounting for over 60% of the study sample. Women are the caregivers in most cases and usually have good health-seeking behaviour as compared with men.^3^ Women are also caregivers of under- fives, and they are bound to utilise facilities more as compared with men.^2^ This finding was consistent in the two provinces. There is a need that health care facilities try and promote male involvement by offering more male-engaging programmes to improve their participation.

The majority of respondents (over 86%) in FS walk to the health facilities as compared with GP with about 65% of the respondents walking. Therefore, more money (about 3.6 rand per trip) is spent on transport in GP as compared with FS (about 1 rand per trip) as most people would tend to walk to the health facilities in FS and in turn utilise facilities that are closer to them, that is, from their catchment. Other authors argue that FS is largely rural and has poor road networks as compared with GP prompting more patients in FS to walk to the nearest clinics that are even 30 minutes away from their homesteads.^24^ There is, therefore, a need for policymakers to ensure that there are adequate facilities in communities in FS as individuals might be deterred from going to facilities as they are far away from them.

Time spent at the clinics, being listened to by nurses, knowing the name of nurse, privacy being respected, being given information on the condition you are suffering from and being treated politely were significantly associated with patient satisfaction in both provinces. These findings are well supported by Dookie and other authors who found that patient interaction with staff members improves the understanding of treatment plans, boosts patients’ morale and improves chances of them abiding to treatment plans and thus better health outcomes.^11,25^ If these factors are not well considered, they can lead to failure of health systems as patients might have unrealistic expectations, which may result in defaulting of treatment through dissatisfaction.^11,25^ There is, therefore, a need for health care providers to improve these factors so as to improve the level of satisfaction.

### Limitations of the study

This study was limited to only two provinces; therefore, the results cannot be generalised to other provinces. There could be patients who were not utilising the PHC services at the time of conducting the primary study who might have been dissatisfied; this could have led to overestimation of patient satisfaction. There is a possibility that patients could be satisfied with a particular facility or service on that particular day, which might have led to negativity or positivity in their responses, which would then not be a true reflection of how satisfied they were with PHC services in general. This study utilised secondary data for analysis; there was no way of rectifying the missing information on some records and variables, and this could have led to some inaccurate inferences. Some variables like culture, socioeconomic status, age and classification on whether respondents were responding in their personal capacities or on behalf of their children could have given a wide range of factors that could have had a bearing on patient satisfaction and could influence differences and similarities in these two provinces. These variables were not measured in the primary study. In the primary study, the exact time spent by patients in these facilities was not ascertained; this was because of the fact that this variable was categorised into three categories, that is, too much, just right and too short. Such categorisation does not give a clear picture of the time spent as it is subjective: what other respondents might consider as too much time might not be interpreted the same by other respondents.

## Conclusion

The findings of this study suggest that high levels of satisfaction with PHC services were experienced by study participants in both provinces. There is, however, a need to address or improve these factors so as to increase levels of patient satisfaction. Satisfied patients adhere to treatment plans and have better health-seeking behaviour, which translates to improved clinical outcomes. Therefore, nurses should continue listening, respecting and treating their patients with politeness and also implement efficient work schedules to reduce patient waiting times. The PHC re-engineering and the rollout of the NHI could leverage on findings from this kind of studies to inform their policies and decisions so as to contextualise their services.

## Appendix 1

### Building the logistic multiple regression

The logistic multiple regressions were conducted in a series of preliminary steps. Firstly, variables were tested in the bivariate analysis for significant association with the outcome variable, that is, ‘respondents encouraging friends and family members to use the same clinic they attended’. Variables having a *p*-value of 0.2 or less in this test were selected for inclusion in the model (it is standard practice to use a greater *p*-value during the initial selection stages so that factors are not lost early during the building of the model as it goes through a series of steps). Forward selection was then used to build the final multiple regression model. The final model had only variables, which remained with a *p*-value of less than 0.05 in the forward model. This enabled comparison of factors within provinces and between provinces to establish whether or not there were differences in factors influencing patient satisfaction with PHC services offered in these two provinces.
